# The Charcot–Marie Tooth Disease Mutation R94Q in MFN2 Decreases ATP Production but Increases Mitochondrial Respiration under Conditions of Mild Oxidative Stress

**DOI:** 10.3390/cells8101289

**Published:** 2019-10-21

**Authors:** Christina Wolf, Rahel Zimmermann, Osamah Thaher, Diones Bueno, Verena Wüllner, Michael K.E. Schäfer, Philipp Albrecht, Axel Methner

**Affiliations:** 1 Institute of Molecular Medicine, University Medical Center, Johannes Gutenberg-Universität Mainz, 55131 Mainz, Germany; wolf.christina90@gmail.com (C.W.); zimmermann.rahel@gmail.com (R.Z.); osamah.thaher@gmail.com (O.T.); diones.bueno@gmail.com (D.B.); verena.wuellner@gmail.com (V.W.); 2 Department of Anesthesiology, Research Center for Immunotherapy (FZI), Focus Program Translational Neurosciences (FTN), University Medical Center, Johannes Gutenberg-Universität Mainz, 55116 Mainz, Germany; michael.schaefer@unimedizin-mainz.de; 3 Department of Neurology, University Hospital Düsseldorf, 40210 Düsseldorf, Germany; phil.albrecht@gmail.com

**Keywords:** oxidative stress, MFN2, mitochondria, fusion/fission

## Abstract

Charcot–Marie tooth disease is a hereditary polyneuropathy caused by mutations in Mitofusin-2 (MFN2), a GTPase in the outer mitochondrial membrane involved in the regulation of mitochondrial fusion and bioenergetics. Autosomal-dominant inheritance of a R94Q mutation in MFN2 causes the axonal subtype 2A2A which is characterized by early onset and progressive atrophy of distal muscles caused by motoneuronal degeneration. Here, we studied mitochondrial shape, respiration, cytosolic, and mitochondrial ATP content as well as mitochondrial quality control in MFN2-deficient fibroblasts stably expressing wildtype or R94Q MFN2. Under normal culture conditions, R94Q cells had slightly more fragmented mitochondria but a similar mitochondrial oxygen consumption, membrane potential, and ATP production as wildtype cells. However, when inducing mild oxidative stress 24 h before analysis using 100 µM hydrogen peroxide, R94Q cells exhibited significantly increased respiration but decreased mitochondrial ATP production. This was accompanied by increased glucose uptake and an up-regulation of hexokinase 1 and pyruvate kinase M2, suggesting increased pyruvate shuttling into mitochondria. Interestingly, these changes coincided with decreased levels of PINK1/Parkin-mediated mitophagy in R94Q cells. We conclude that mitochondria harboring the disease-causing R94Q mutation in MFN2 are more susceptible to oxidative stress, which causes uncoupling of respiration and ATP production possibly by a less efficient mitochondrial quality control.

## 1. Introduction

Charcot–Marie tooth (CMT) disease is one of the most common inherited peripheral neuropathies with a prevalence rate of 1/2500. It is clinically characterized by progressive muscle weakness and atrophy and can be classified by electrophysiological and histological criteria as demyelinating (CMT 1) or axonal (CMT 2) [1]. Mutations in the protein Mitofusin-2 (MFN2), a GTPase of the outer mitochondrial membrane involved in mitochondrial fusion, causes the axonal subtype 2A which can be inherited in an autosomal-dominant and recessive manner. Regardless of the inheritance pattern or the clinical manifestation of the peripheral neuropathy, the testing for *MFN2* gene mutations has been recommended as a first-line analysis in the axonal subtype [2].

The vital role of MFN2 or its close homologue MFN1 has been demonstrated in mice deficient in either of these mitofusins as it results in embryonic lethality during mid-gestation [3]. MFN2 not only controls fusion of the outer mitochondrial membrane [4] but also plays a critical role in the metabolic functions of mitochondria. Suppression of MFN2 expression reduces mitochondrial membrane potential, cellular respiration and mitochondrial proton leak [5]. Conversely, overexpression of MFN2 increased cellular respiration even when expressed as a fusion-inactive deletion mutant [6]. Others, including us, found increased cellular respiration in MFN2-deficient mouse embryonic fibroblasts (MEFs) compared to wildtype controls [7,8] and we recently reported that these discrepancies might be due to differences in redox conditions sensed by the thiol switch cysteine 684 [8]. MFN2 is also a key determinant of so-called mitochondria-endoplasmic reticulum (ER) contact sites (MERCS), also known as mitochondria-associated membranes (MAMs), which represent hot spots of interactions and serve as important signaling hubs between these cellular organelles. MFN2 occurs on both sides of MAMs and apparently tethers the ER to mitochondria by homo- and heterotypic complexes with itself or its homologue MFN1 [9]. This traditional view that lack of MFN2 loosens ER-mitochondria interaction and thereby mitigates the inositol-1,4,5-trisphosphate (IP3) receptor dependent Ca^2+^ flux from the ER to mitochondria [9], has, however, recently been challenged. By using elaborate electron microscopy techniques, Cosson et al. found increased ER-mitochondria juxtaposition in MFN2-deficient cells [10], thus the opposite of what was previously thought. This was later reproduced using a whole array of different techniques and it was concluded that MFN2 rather works as a tethering antagonist preventing an excessive, potentially toxic proximity between the two organelles [11]. However, even this was challenged and reduced levels of the mitochondrial Ca^2+^ uniporter (MCU) were introduced as an additional complicating factor [12]. It is probably safe to conclude that MFN2 is involved in MAM formation and integrity.

Züchner et al. were the first to identify a heterozygous 281G-A transition in the *MFN2* gene in a Russian kindred with CMT2A2A with an age of disease onset between 3 and 17 years [13]. This mutation results in an arginine 94 to glutamine (R94Q) substitution in a helix bundle preceding the GTPase domain of the protein. Transgenic mice expressing this mutation in human MFN2 develop locomotor impairments and gait defects. This phenotype coincided with distal axon accumulation of mitochondria in the sciatic nerve [14] and mitochondrial respiratory chain defects of complexes II and V associated with a drastic decrease of ATP synthesis [15]. Using the same model and elaborate techniques to quantify mitochondrial ATP and hydrogen peroxide in resting or stimulated peripheral nerve myelinated axons in vivo, it was recently demonstrated that R94Q mitochondria fail to match the increased demand of ATP production in stimulated axons whereas the production of H_2_O_2_ was almost unaffected. The authors concluded that neuropathic conditions uncouple the production of reactive oxygen species (ROS) and ATP, thereby potentially compromising axonal function and integrity [16]. Finally, R94Q MFN2 appears to lead to reduction in MERCS both in CMT2A patient-derived fibroblasts and primary neurons in vitro and in vivo in motoneurons of the above-mentioned mouse model of CMT2A [17]. This was associated with increased ER stress, defective Ca^2+^ handling, and alterations in the geometry and axonal transport of mitochondria [17].

In this contribution, we studied mitochondrial shape, respiration, and mitochondrial quality control in MFN2-deficient fibroblasts stably expressing wildtype or R94Q MFN2. We found that mild oxidative stress induced by 24 h pretreatment with 100 µM hydrogen peroxide significantly increased respiration but decreased mitochondrial ATP generation in R94Q—but not in wildtype cells. This coincided with a defective PINK1/Parkin-mediated mitophagy. Our results suggest that the disease-causing R94Q mutation in MFN2 uncouples mitochondrial respiration from ATP production by a less efficient mitochondrial quality control under conditions of mild oxidative stress.

## 2. Materials and Methods

### 2.1. Cell Culture

Cell culture experiments were carried out with Mfn2^−/−^ and Mfn2^+/+^ MEF [3] kindly provided by Timothy Shutt (University of Calgary). Cells were grown according to standard methods under controlled conditions using in DMEM High Glucose (Sigma-Aldrich) supplemented with 10% (*v/v*) fetal calf serum (FCS, Thermo Scientific, Waltham, MA, USA), 100 U/mL penicillin and 100 µg/mL streptomycin (Thermo Scientific, Waltham, MA, USA) at 37 °C in a humidified incubator (5% CO_2_). The PiggyBac system was used to create cells stably expressing HA-MFN2-IRES-mCherry-NLS constructs followed by enrichment of mCherry-positive cells using fluorescence-activated cell sorting essentially as described [18].

### 2.2. Immunoblotting

Cell were lysed with RIPA buffer and subjected to SDS-Page and immunoblotting according to standard methods essentially as described [8,19]. Primary antibodies were anti-MFN2 mAB (1:500; Abnova, Taipei City, Taiwan), anti-G6PD (1:1000; Cell Signaling, Danvers, MA, USA), anti-PKM1/2 (1:1000; Cell Signaling), anti-Hexokinase I (1:1000; Cell Signaling), anti-Hexokinase II (1:1000; Cell Signaling) and anti-Actin mAB (1:4000; Merck Millipore, Burlington, MA, USA). Protein bands were revealed and analyzed following incubation for 1 h at room temperature with a secondary goat anti-mouse IgG antibody conjugated to an infrared fluorescent dye (IRDye 800, Licor, Bad Homburg, Germany) using the Odyssey near infrared laser imaging system (Licor, Bad Homburg, Germany). For mitophagy induction, cells were treated with 10 µM carbonyl cyanide 3-chlorophenylhydrazone (CCCP) for the indicated time under normal culturing conditions. The reaction was stopped by washing steps with PBS and cell lysis with RIPA buffer.

### 2.3. Measurement of Mitochondrial Oxygen Consumption

Intact MEF cells were monitored for mitochondrial oxygen consumption using a high-resolution respirometer (Oxygraph-2k, Oroboros Instruments, Innsbruck, Austria) as previously described [8,19] using identical substrates and inhibitors purchased from Sigma-Aldrich (St. Louis, MO, USA). Briefly, after recording routine respiration, ATP synthase activity was inhibited by 2.5 µM oligomycin to determine leak respiration. Stepwise addition of 0.5 µM carbonyl cyanide 4-(trifluoromethoxy) phenylhydrazone (FCCP) was performed to reveal the maximum capacity of the electron transfer system (ETS). Non-mitochondrial residual oxygen consumption (ROX) was determined following inhibition of respiration by application of 0.5 µM rotenone and 2.5 µM antimycin A. Mitochondrial respiration changes in response to redox alterations were examined after 24 h pretreatment of cells with 100 µM hydrogen peroxide (H_2_O_2_)_._ All experiments were carried out after correction of instrumental background and calibration of the polarographic oxygen sensors. Data analysis was done using the DatLab Software 5.1 (Oroboros Instruments, Innsbruck, Austria) as described [8,19] and all values were corrected for ROX and instrumental background.

### 2.4. Cell Proliferation Assay

MEF cells were seeded into white 96-well plates at a density of 750 cells/well. The same day, NanoLuc luciferase and substrate (G9711, Promega, Madison, WI, USA) were added simultaneously to the cell culture media. Metabolically active cells can reduce the substrate, which in turn can react with the luciferase. Total luminescence was directly measured using the Infinite 2000 Pro microplate reader (Tecan, Männedorf, Switzerland). After 24 h, the luminescence was measured a second time and the cells were treated with 100 µM H_2_O_2_. The luminescence was measured again after 24 h and 48 h. Data are expressed as proliferation rate normalized to the first day (proliferation rate = 0).

### 2.5. Quantification of ATP Levels

Relative ATP levels were determined with BTeam, a BRET-based ATP biosensor [20]. MEF cells were seeded into white 96-well plates at a density of 2000 cells/well and transfected 24 h later using TurboFectin reagent (OriGene, Rockville, MD, USA) with BTeam lacking a targeting sequence (for cyto-ATP determination) or containing a mitochondrial targeting sequence (for mito-ATP determination). After 24 h, cells were treated with H_2_O_2_ for additional 24 h and then incubated for 30 min in phenol red-free DMEM containing 10% FBS, and 30 µM NanoLuciferase (NLuc) inhibitor to prevent unintended detection of BTeam released from dead cells. Cells were subsequently incubated for 20 min in the presence of NLuc substrate (Promega, Madison, WI, USA) and the luminescence was measured at 520/60 nm (ex/em) (Yellow Fluorescent Protein (YFP) emission) and at 430/70 nm (ex/em) (NLuc emission) at 37 °C. Data are expressed as YFP/NLuc emission ratio.

### 2.6. Measurement of Total Cellular GSH

Cells were plated in a 6-well plate at a density of 200,000 cells/well, treated 24 h later for two different time periods (2 h and 24 h) with 100 µM H_2_O_2_. Cells were washed twice with ice-cold PBS and resuspended in 200 µL SSA/HCl buffer containing 1.3% *w/v* sulfosalicylic acid and 8 mM HCl in KPE buffer. For KPE buffer, 0.1 M solutions of KH_2_PO_4_ and K_2_HPO_4_ were prepared; 16 mL and 84 mL of these solutions were mixed respectively with 5 mM EDTA in order to obtain 100 mL of a 0.1 M phosphate buffer with a pH of 7.5. Samples were vortexed and incubated on ice for 10 min and centrifuged at 14,000 rpm for 10 min. The pellet was resuspended in 0.2 N NaOH and incubated at 37 °C overnight, followed by protein quantitation (BC Assay, Interchim). The supernatant was split into two new microcentrifuge tubes containing 12 µL of triethanolamine/H_2_O 1:1, for GSH and GSSG quantification. 2 µL of 2-Vinylpyridine (2-VP, Sigma-Aldrich, prediluted 1:5 in EtOH) was added to the GSSG tubes and incubated on ice and in the dark for one hour. GSH was then measured by monitoring NADPH consumption by GSH reductase in KPE assay buffer, containing 2.8 mM DTNB (5,5′-dithiobis-(2-nitro-benzoic acid)) and 1.3 mM NADPH, at 390 nm using the Infinite 2000 Pro microplate reader (Tecan, Männedorf, Switzerland) and the SpectraMax I3 microplate reader. The same assay procedure was carried out for the 2-VP treated GSSG samples and the final GSH/GSSG concentrations were normalized to the total protein amount. All chemicals were obtained from Sigma-Aldrich (St. Louis, MO, USA).

### 2.7. Lactate Measurement

Cells were plated in a 6-well plate at a density of 200,000 cells/well and treated after 24 h with H_2_O_2_ for additional 24 h. A minimum of 200 µL of the culture medium was removed for lactate quantification, which was performed by using the Alinity Lactic Acid Reagent Kit (8P2120, Abbott, Chicago, IL, USA) following the manufacturers’ instructions. The reaction was measured photometrically with the Alinity c analyzer (Abbott, Chicago, IL, USA). Measured lactate concentrations were normalized to the total protein amount per well.

### 2.8. RNA Isolation and PCR

Cells were treated with 100 µM H_2_O_2_ for 24 h. RNA of cells was prepared using the ZR RNA MiniPrep™ kit from Zymo Research (R1064, Irvine, CA, USA) following manufacturers’ instructions. RNA was reversed transcribed using the cDNA synthesis kit (Life Technologies, 4368814). Quantitative real-time PCR was performed with the 7500 Real-Time PCR Systems device (Applied Biosystems, Foster City, CA, USA) using FAM/Dark quencher probes from the Universal Probe Library™ (Roche, Basel, Switzerland). The expression was quantified to the relative levels of the housekeeping gene hypoxanthineguanine phosphoribosyltransferase (HPRT), which was assessed by FAM-TAMRA probe FAM-CCTCTCGAAGTGTTGGATACAGGGCA-TAMRA and the primers forward GTTGCAAGCTTGCTGGTGAA and reverse GATTCAAATCCCTGAAGTACTCA. For amplification and detection of glutamate-cysteine ligase, catalytic subunit (GCLc), glutathione S transferase omega 1 (GSTO1), NADPH-quinone-oxidoreductase-1 (NQO1), glutathione peroxidase 1 (GPX1), heme-oxygenase-1 (HO1) and cystine/glutamate transporter (xCT) the following primers were used: xCT: forward TGGGTGGAACTGCTCGTAAT, reverse AGGATGTAGCGTCCAAATGC, probe 1 (Universal Probe Library™, Roche). GCL forward GGAGGCGATGTTCTTGAGAC, reverse CAGAGGGTCGGATGGTTG probe 2 (Universal Probe Library™, Roche). GST1 forward CAGCGATGTCGGGAGAAT, reverse GGCAGAACCTCATGCTGTAGA, probe 60 (Universal Probe Library™, Roche). GPX1: forward TTTCCCGTGCAATCAGTTC, reverse TCGGACGTACTTGAGGGAAT, probe 2 (Universal Probe Library™, Roche). HO1: forward GTCAAGCACAGGGTGACAGA, reverse ATCACCTGCAGCTCCTCAAA, probe 4 (Universal Probe Library™, Roche). NQO1: forward AGCGTTCGGTATTACGATCC, reverse AGTACAATCAGGGCTCTTCTCG, probe 50 (Universal Probe Library™, Roche, Basel, Switzerland). RNAse-free water was used as the non-template control. Analysis of the results was performed using the ΔΔCT-method. All conditions were normalized to their untreated control group.

### 2.9. Microscopy and Image Analysis

For microscopic image analysis, cells were plated into 8-well-glass-bottom slides (IBIDI, Gräfelfing, Germany) to reach a final cell confluency of 60–80% on the day of image acquisition. Cells were exposed to 100 µM H_2_O_2_ or vehicle 24 h before image recording. Image acquisition was done with the confocal microscope Leica TCS SP5 (Leica Microsystems, Wetzlar, Germany) using a 63× oil immersion objective. For mitochondrial morphology analysis, cells were seeded as described above, cultured for 24 h in serum-supplemented medium and incubated for 15 min with 0.2 μM MitoTracker Red CMXRos (Invitrogen, Carlsbad, CA, USA) in serum-free medium. Cells were washed once with PBS (Sigma-Aldrich) and further incubated for 15 min in serum-supplemented medium. Live cell imaging was performed with an ex/em of 560/610 nm. Cells were categorized according to their mitochondrial morphology (tubular, mixed or fragmented) and analyzed by observers blind to the experimental conditions and genotypes.

Cellular ROS was analyzed by staining with the CellROX reagent (Molecular Probes, Eugene, OR, USA) in a final concentration of 5 µM for 30 min at 37 °C. After thoroughly washing with PBS, pre-warmed DMEM without phenol red (Thermo Scientific) was added and the fluorescence intensity was measured at 485/520 nm (ex/em). For glucose uptake measurement, cells were plated into 8-well-glass-bottom slides (IBIDI, Gräfelfing, Germany). Next day, cells were treated with 100 µM H_2_O_2_ or vehicle for 24 h before cells were incubated with the fluorescent d-glucose analog 2-[N-(7-nitrobenz-2-oxa-1,3-diazol-4-yl) amino]-2-deoxy-d-glucose (2-NBDG) (Thermo Scientifc) at a final concentration of 100 µM for 60 min at 37 °C. After washing with PBS for two times, pre-warmed DMEM without phenol red (Thermo Scientific) was added and the fluorescence intensity was measured at 465/540 nm (ex/em) and analyzed with ImageJ (Fiji). Transfections were performed with Lipofectamine 2000 reagent (Thermo Scientific) according to manufacturers’ instructions. Briefly, 100 µL and 125 µL Opti-MEM (Gibco Life Technologies, Waltham, MA, USA) was mixed with 8 µL Lipofectamine and 2.5 µg DNA, respectively. Subsequently, 90 µL of each solution was mixed and incubated for 5 min at room temperature before 10 µL was added to each well. Image recording was carried out 48 h after transfection. Cells were transfected with Mt-Keima (kind gift of Atsushi Miyawaki, RIKEN Brain Science Institute, Japan) or Mito-Timer (Addgene, Watertown, NY, USA). 24 h after H_2_O_2_ treatment for Mt-Keima the intensities of the fluorescence at 620 nm were captured when excited at 550 nm and 438 nm. The intensity was analyzed using ImageJ (Fiji), selecting the cytoplasm of the cells using regions of interest outside the nucleus and then analyzing the mean intensity. The intensity at 550 nm was normalized to the intensity at 438 nm. For Mito-Timer, fluorescence intensity was measured at 561/572 nm (ex/em) and 488/518 nm (ex/em). For measurement of the colocalization of GFP-Parkin (kind gift of Julia Fitzgerald, University of Tübingen, Germany) and Mito-TurboFarRed the fluorescence intensity was measured at 488/518 nm (ex/em) for GFP-Parkin and at 633/640 nm (ex/em) for Mito-TurboFarRed. Parkin-Mito-TurboFarRed colocalization was measured using the JACoP plugin of ImageJ (Fiji).

### 2.10. Statistical Analyses

Statistical analyses were conducted using GraphPad Prism (GraphPad). The respective statistical tests are mentioned in the figure legends. Statistically significant differences were assumed with *p* < 0.05.

## 3. Results

### 3.1. More Fragmented Mitochondria but Similar Basal Respiration in Cells Expressing Wildtype and Disease-Causing R94Q MFN2

**Figure 1:**
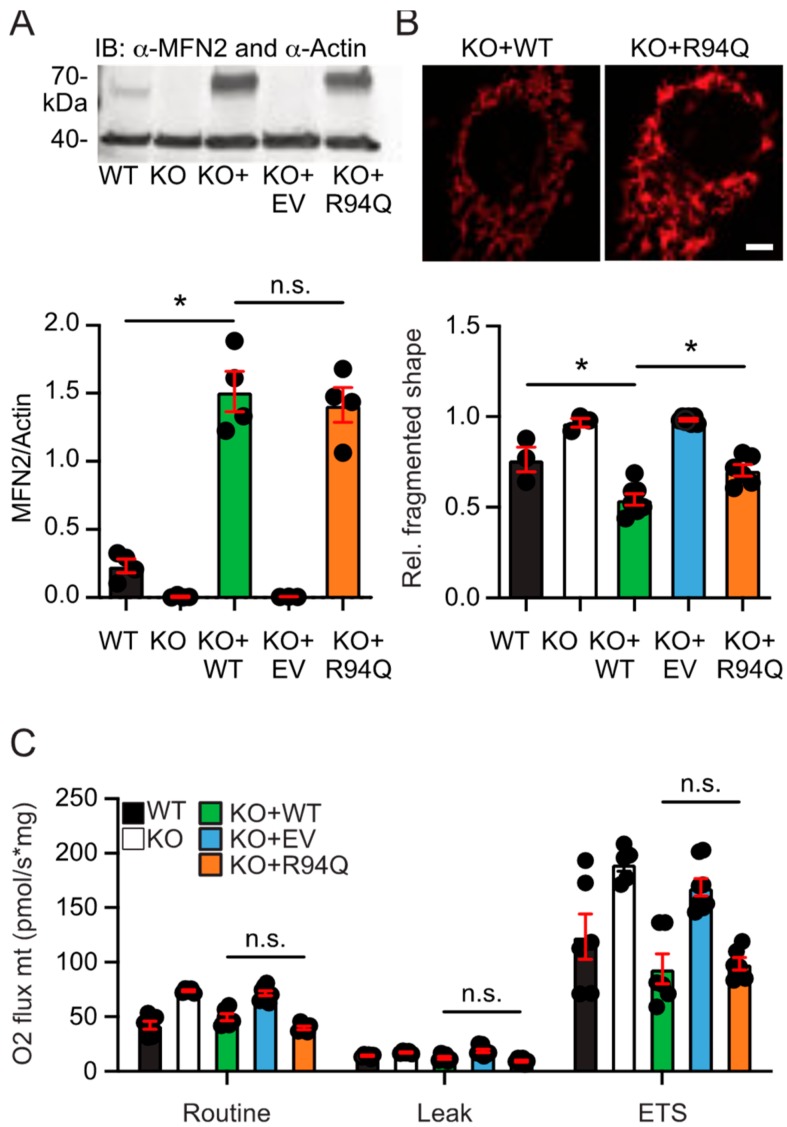
Fragmented mitochondria but similar basal respiration in cells expressing wildtype and disease-causing R94Q MFN2. (**A**) Immunoblot showing endogenous MFN2 in wildtype (WT) cells and similar MFN2 protein overexpression levels in MFN2 knockout (KO) cells rescued with wildtype MFN2 (KO + WT) or R94Q MFN2 (KO + R94Q). Actin served as loading control. (**B**) Increased mitochondrial fragmentation in KO + R94Q cells. Mitotracker-stained cells were categorized upon their mitochondrial morphology as tubular, mixed, or fragmented by blinded investigators. A representative picture of KO + WT and KO + R94Q is shown. (**C**) Oxygen flow per cells quantitated by high-resolution respirometry demonstrates similar routine, leak and electron transfer system capacity (ETS) of KO + WT and KO + R94Q cells. Data are expressed as mean ± SEM of n = 4 immunoblots in (**A**), n = 6 independent experiments analyzed by two blinded investigators in (**B**), and n = 5 independent experiments in (**C**). Statistical significance was determined using one-way ANOVA and Tukey’s multiple comparison test (* *p* < 0.05; n.s., non-significant).

We generated cells stably expressing wildtype (WT) or R94Q MFN2 in MFN2 knockout MEFs [3] to study the effect of the disease-causing mutation R94Q on mitochondrial shape and function. To avoid potentially toxic antibiotics such as puromycin or G418, which are often used for the generation of stable cell lines but might affect cellular physiology, we enriched cells expressing HA-tagged WT and mutant MFN2 inserted into the genome by Piggybac-mediated gene transfer using several rounds of fluorescence-activated cell sorting. All stably transfected cells expressed the red fluorescent mCherry reporter protein targeted to the nucleus downstream of an internal ribosomal entry site (IRES). Cells expressing an additional mCherry before the IRES-mCherry served as negative control (EV, empty vector). Both cell lines, WT and R94Q, expressed similar levels of MFN2 shown by immunoblotting (Figure 1a). Analyzing >100 mitotracker-stained cells classified by blinded investigators into cells containing fragmented, tubular or mixed mitochondria demonstrated a significantly more fragmented shape of mitochondria in KO + R94Q compared to KO + WT cells (Figure 1b). We then assessed mitochondrial respiration of intact cells in culture medium by high-resolution respirometry assessing routine and leak respiration as well as the maximal electron transfer capacity (ETS). Leak respiration was recorded after addition of the ATP synthase inhibitor oligomycin and the ETS by titrating the uncoupler and protonophore FCCP as described [21]. Non-mitochondrial oxygen consumption, ROX, which represents oxygen consumption of auto-oxidation reactions and other cellular oxygen-consuming enzymes such as oxidase and peroxidase [21] was recorded after inhibition of complex I with rotenone and of complex III with antimycin A and subtracted from the routine, leak or ETS values. We observed that oxygen flow per cell at routine respiration and the ETS was significantly increased in KO cells and in KO cells expressing EV as previously reported [7,8] whereas overexpression of both WT and R94Q MFN2 similarly rescued the respiration phenotype to levels indistinguishable from the parental wildtype cells (Figure 1c). We therefore detected no differences between WT and R94Q-expressing cells in mitochondrial respiration under normal culture conditions.

### 3.2. Mild Oxidative Stress Causes Increased Mitochondrial Fragmentation and Uncoupling in Cells Expressing R94Q MFN2

**Figure 2:**
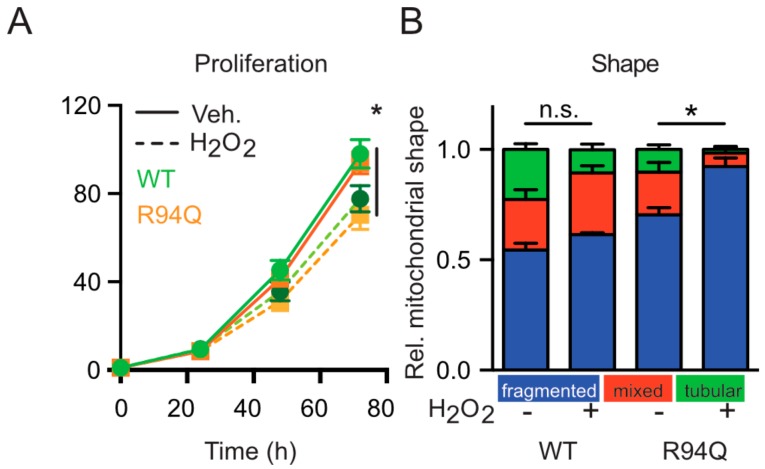
Oxidative stress causes increased mitochondrial fragmentation in cells expressing R94Q MFN2. (**A**) Reduced but similar proliferation rate of WT and R94Q cells exposed to H_2_O_2_. Similar amounts of cells were seeded and treated with 100 µM H_2_O_2_ 24 h later. Luminescence was measured every 24 h for 72 h. Data are expressed as mean proliferation rate ± SEM of n = 4 independent experiments done in triplicates and normalized to the first day. (**B**) Relative distribution of mitochondrial morphologies in mitotracker-stained cells. Cells were categorized according to their mitochondrial morphology as tubular, mixed, or fragmented. Data are expressed as mean ± SEM of n = 4 independent experiments analyzed by investigators blind to cell line identity in (**B**). Statistical significance was determined using one-way ANOVA and Tukey’s multiple comparison test (* *p* < 0.05; n.s., non-significant).

We figured that a potential disease-related phenotype might be revealed by stressing the cells and subjected the cells to conditions of mild oxidative stress induced by low levels of hydrogen peroxide (100 µM) added once 24 h before analysis. This concentration corresponds to an intracellular concentration of 1 µM [22] and did not compromise cell survival as examined by microscopy but slightly slowed down the cellular proliferation rate without differences between WT and R94Q cells (Figure 2a). The treatment however further increased the number of cells with fragmented mitochondria which was more pronounced in the R94Q cells (Figure 2b) suggesting that the disease-causing mutation renders the mitochondria more susceptible to oxidative stress.

**Figure 3:**
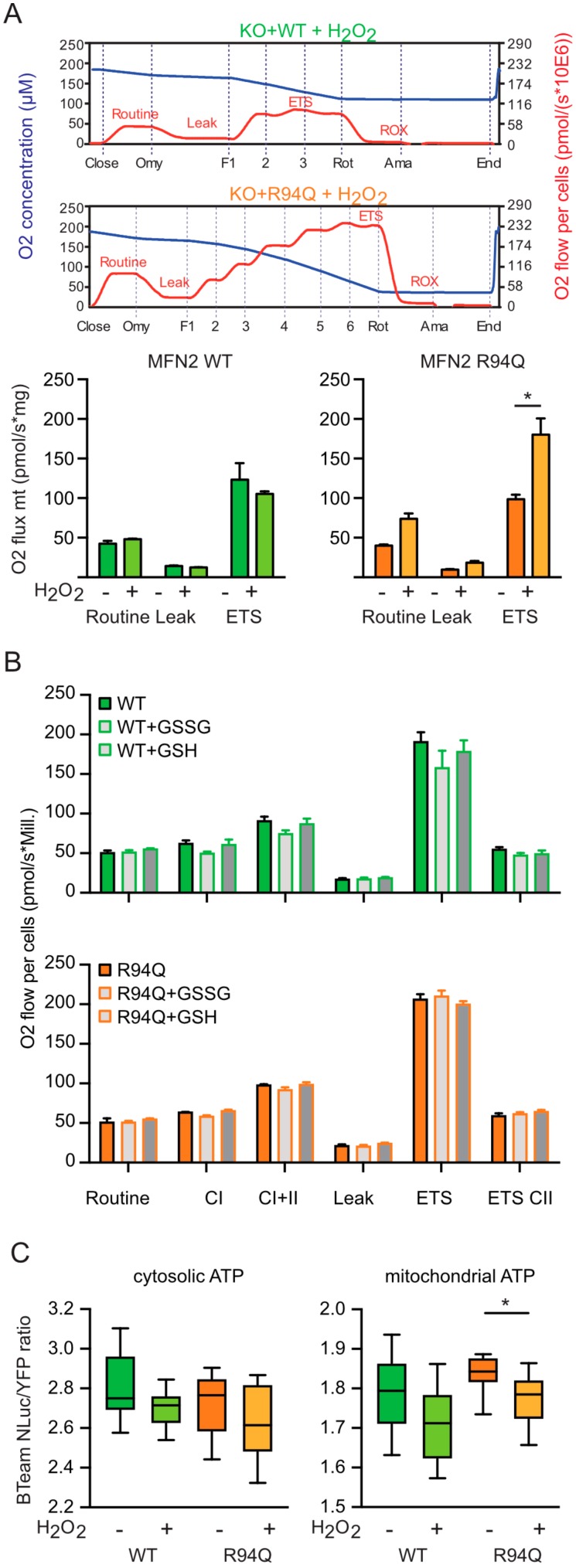
Oxidative stress causes increased mitochondrial uncoupling in cells expressing R94Q MFN2. (**A**) Representative high-resolution respirometry of intact cell recordings showing oxygen concentration (blue line) and oxygen flow per cells (red line) over time. Time points of oligomycin (Omy), FCCP (F), rotenone (Rot) and antimycin A (Ama) additions are indicated. Oxygen flow per cells were corrected for ROX at the indicated mitochondrial respiration state. Basal cellular routine respiration, leak and ETS capacity increased in R94Q but not WT treated with 100 µM H_2_O_2_ 24 h prior to measurement. (**B**) High-resolution respirometry of cells permeabilized with digitonin with GSH or GSSG added. Mitochondrial respiration stimulated by ADP represents complex I (CI) activity, addition of succinate and ADP corresponds to respiration with convergent input of electrons via complexes I and II into the respiratory system (CII). Mitochondrial membrane integrity was tested by the application of cytochrome c. ATP synthase inhibition by oligomycin revealed the leak state (Leak). The electron transfer system (ETS) capacity at maximum oxygen flow per cells was determined by titration of FCCP and ROX after antimycin A-induced inhibition of complex III. Data are expressed as mean oxygen flow per cell corrected for ROX ± SEM at the indicated mitochondrial respiration state. GSH and GSSG had no effect on mitochondrial respiration. Data in A and B show the mean ± SEM of n = 6 independent experiments. Statistical significance was determined using multiple *t*-tests with a false discovery rate (Q) of 1% according to the two-stage method by Benjamini, Krieger and Yekutieli (* *p* < 0.05; n.s., non-significant). (**C**) Relative ATP levels quantitated by targeting BTeam, a ratiometric BRET-based ATP biosensor to the cytosol or to the mitochondrial matrix. Data are expressed as YFP/NLuc emissions ratio. Statistical variation in (**C**) is shown as Tukey boxplots and significance calculated using student’s *t*-test comparing cell lines with and without H_2_O_2_ exposure, * *p* < 0.05, n = 4 independent experiments done in triplicates.

To clarify the functional consequences on mitochondrial respiration, we next compared potential alterations in mitochondrial respiration upon hydrogen peroxide exposure. Interestingly, hydrogen peroxide had no significant effect on routine, leak and ETS respiration in WT cells but greatly and significantly increased all parameters in R94Q-expressing cells (Figure 3a). As this very much resembled the phenotype we recently described for KO cells stably transfected with the mutant C684A, a mutant that removes a cysteine involved in the process of mitochondrial hyperfusion [23], we repeated a key experiment of this publication which demonstrated that lack of cysteine 684 increased MFN2 susceptibility to environmental redox alterations. In these experiments, we found that treating digitonin-permeabilized cells with 1 mM GSH for 10 min greatly reduced respiration in C684A but not in WT MFN2 cells [8]. To clarify whether R94Q has the same effect, we repeated this experiment and studied mitochondrial respiration in the presence or absence of GSH and GSSG in cells permeabilized with digitonin. This, however, revealed no difference between WT and R94Q cells (Figure 3b) suggesting that the similar increase in respiration of C684A [8] and R94Q upon exposure to 100 µM H_2_O_2_ must be caused by a different mechanism. We next assessed whether this increase in respiration corresponds to an increased ATP production and transfected the genetically encoded reporter BTeam [20] targeted to mitochondria or the cytosol into our cells. BTeam measures ATP by bioluminescence resonance energy transfer (BRET) and is ratiometric, making comparison of different cell lines feasible. Using this approach, we found no difference between the cell lines at steady state but a significant drop in mitochondrial ATP after H_2_O_2_ exposure only in R94Q cells (Figure 3c). From these experiments, we concluded that mild oxidative stress causes mitochondrial uncoupling of respiration and ATP production in cells expressing the disease-causing R94Q mutation.

### 3.3. Rather Reduced and Not Increased Oxidative Stress in Cells Expressing R94Q MFN2

**Figure 4:**
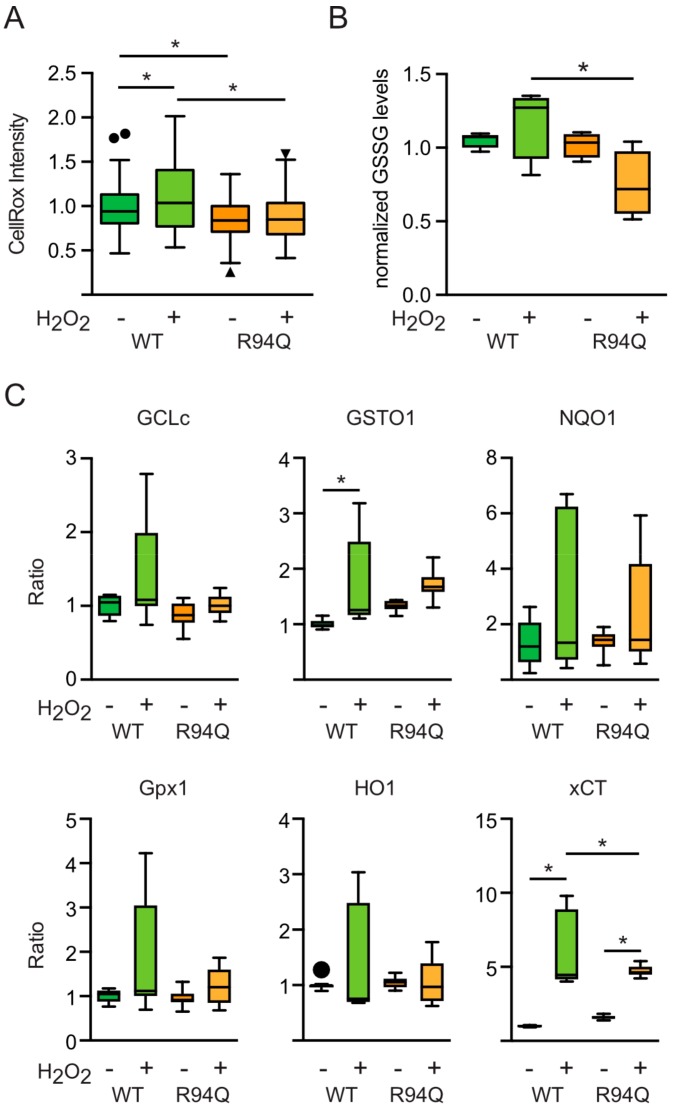
Reduced and not increased oxidative stress in cells expressing R94Q MFN2. (**A**) Cells were stained with CellRox and the intensity in single cells quantitated by confocal microscopy. (**B**) GSSG was measured by monitoring NADPH consumption by GSH reductase in 2-vinylpyridine-treated samples. (**C**) The mRNA of transcripts involved in the antioxidant response were quantified by real-time RT-PCR using Taqman primer-probe assays for glutamate-cysteine ligase, catalytic subunit (GCLc), glutathione S transferase omega 1 (GSTO1), NADPH-quinone-oxidoreductase-1 (NQO1), glutathione peroxidase 1 (GPX1), heme-oxygenase-1 (HO1) and xCT and normalized to the expression of the housekeeping gene hypoxanthine-guanine phosphoribosyltransferase (hprt). RNAse-free water was used as non-template control. Analysis of the results was performed using the ΔΔCT-method. All conditions were normalized to their untreated control group. In all experiments, H_2_O_2_ was added 24 h before analysis. Statistical variation is shown as Tukey boxplots and significance calculated using one-way ANOVA and Tukey’s multiple comparisons test, * *p* < 0.05, in (**A**) n = 3 independent experiments with a total of 150–170 individual cells, in (**B**) n = 5 independent experiments, in (**C**) n = 3 independent experiments done in triplicates.

We reckoned that these changes were provoked by an already higher level of oxidative stress in R94Q cells or by a larger increase upon treatment with hydrogen peroxide. To investigate this, we quantitated reactive oxygen levels at baseline and 24 h after 100 µM H_2_O_2_ using CellRox, a membrane-permeable dye that greatly increases in fluorescence upon oxidation. This, surprisingly, revealed that the untreated R94Q cells had less ROS levels than their WT counterparts (Figure 4a). Also, hydrogen peroxide increased ROS levels in WT but not in R94Q cells (Figure 4a). As a second readout, we quantitated oxidized GSH (GSSG) levels in these cells. This, again, demonstrated a trend toward increased GSSG levels upon treatment in WT in line with the increase in CellRox-quantitated ROS levels in WT cells (Figure 4b). GSSG levels were reduced in treated R94Q cells when compared with treated WT cells (Figure 4b). These unexpected results suggested differences in the antioxidant response between WT and R94Q cells. A major component of the cellular antioxidant response is regulated by transcriptional mechanisms involving the up-regulation of genes with antioxidant response elements (ARE) in their promoters [24]. We therefore quantitated the expression levels of several such genes including glutamate-cysteine ligase, catalytic subunit (GCLc), glutathione S transferase omega 1 (GSTO1), NADPH-quinone-oxidoreductase-1 (NQO1), glutathione peroxidase 1 (GPX1), heme-oxygenase-1 (HO1) and xCT [24,25] at the mRNA level using quantitative RT-PCR. This revealed overall no major differences between the cell lines (Figure 4c); GSTO and the cystine/glutamate antiporter xCT (also known as SLC7A11) were induced in H_2_O_2_-treated cells but at least for xCT significantly less in R94Q cells again in line with a rather reduced oxidative stress in these cells. Therefore, contrary to our expectations, the increased respiratory activity of R94Q cells under conditions of mild oxidative stress could not be explained by basal differences in oxidative stress or a dysregulated antioxidant response. It is possible that the cells expressing mutant MFN2 have up-regulated antioxidant enzymes that keep the oxidant levels low under the conditions studied. These enzymes are either not those investigated here using quantitative RT-PCR or the up-regulation is only apparent at the protein level.

### 3.4. Increased Glucose Uptake and Fueling of Mitochondria with Pyruvate in R94Q Cells

**Figure 5:**
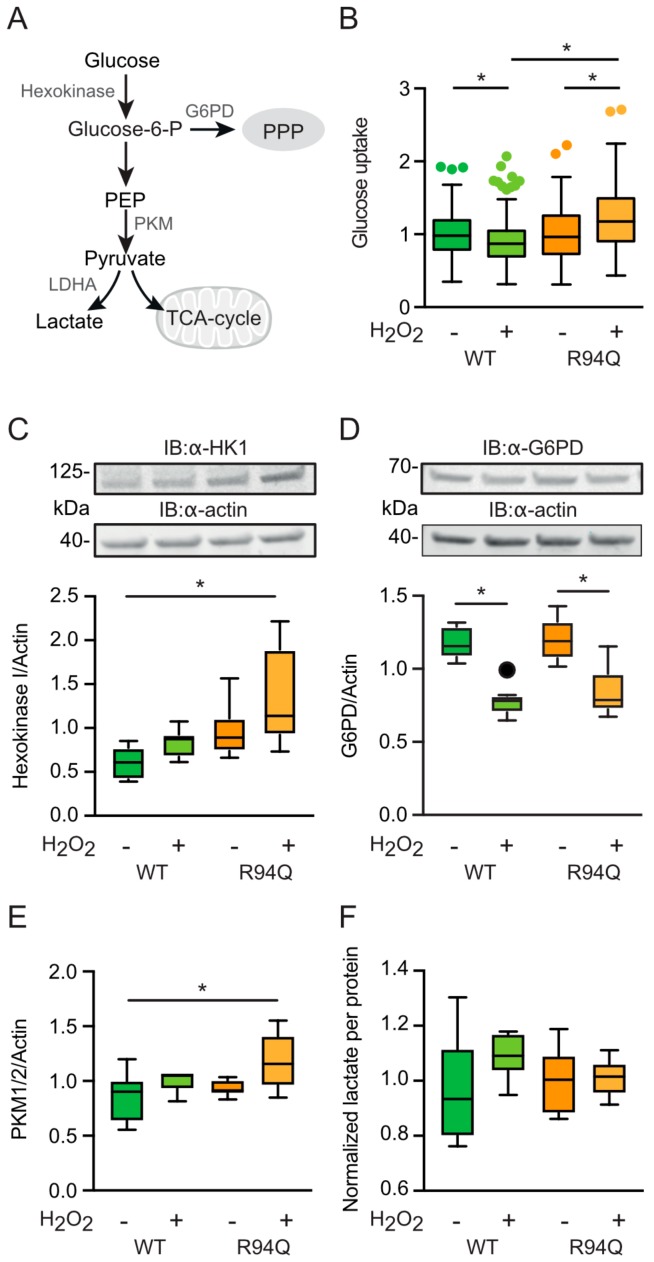
Glucose uptake and fueling of mitochondria with pyruvate in R94Q cells. (**A**) Scheme depicting metabolism upstream of the tricarboxylic acid cycle (TCA). G6PD, glutamate 6-phosphate dehydrogenase; PPP, pentose phosphate pathway; PEP, phosphoenolpyruvate; PKM, pyruvate dehydrogenase isozyme M; LDHA, lactate dehydrogenase A. (**B**) Cells were stained with 2-NBDG and the intensity in single cells quantitated by confocal microscopy. (**C**–**e**) Cells were treated with 100 µM H_2_O_2_ 24 h prior to immunoblotting against (**C**) hexokinase 1 (HK1), (**D**) G6PD, (**E**) PKM isozymes 1 and 2. Actin served as loading control. Size is indicated. (**F**) Lactate levels were measured photometrically and normalized to the protein content of the wells. Statistical variation is shown as Tukey boxplots and significance calculated using one-way ANOVA and Tukey’s multiple comparisons test, * *p* < 0.05, in (**A**) n = 4 independent experiments with a total of 207–243 individual cells, in (**C**–**E**) n = 2 independent blots with a total of 6 individual lysates, (**F**) n = 6 measurements.

Pyruvate is the main fuel for the mitochondrial tricarboxylic acid cycle (TCA) and is generated from glucose by the consecutive activities of hexokinase and pyruvate kinase (PKM) among other enzymes. Hexokinase generates glucose 6-phosphate which can be turned into 6-phosphogluconolactone in the first step of the pentose phosphate pathway (PPP) through the activity of glucose-6-phosphate dehydrogenase (G6PD) and, instead of entering the TCA, pyruvate generated by PKM can be turned into lactate by lactate dehydrogenase (depicted in Figure 5a). A predominance of the PPP was previously shown by us to play a role in the protection against oxidative stress [19,26]. To study alterations in the metabolism upstream of the TCA cycle in WT and R94Q cells with and without exposure to hydrogen peroxide, we quantitated glucose uptake levels using imaging of the specific dye 2-NBDG, the abundance of hexokinase I (HK1), G6PD and pyruvate kinase PKM1/2 using immunoblotting, and lactate levels using an enzymatic assay. This revealed a surprising decrease in glucose uptake respectively levels in WT cells upon H_2_O_2_ treatment and an increase in R94Q cells (Figure 5b). Hexokinase 1, the housekeeping hexokinase, was also significantly increased in R94Q cells exposed to hydrogen peroxide compared to WT cells (Figure 5c). From the fact that the rate-limiting enzyme of the PPP, G6PD, was down-regulated in both cell lines upon hydrogen peroxide treatment and did not differ between the cell lines (Figure 5d), we concluded that the product of hexokinase 1, glucose 6-phosphate, does not enter the PPP. In contrast, the similar regulation pattern of hexokinase 1 and pyruvate kinase PKM1/2 upon H_2_O_2_ exposure (Figure 5e) without significant differences in lactate levels between the cell lines and conditions (Figure 5f) suggest that the increased pyruvate generated by pyruvate kinase in H_2_O_2_-exposed R94Q cells does not enter glycolysis but instead fuels the mitochondrial TCA.

### 3.5. Reduced Mitophagy in R94Q Cells

**Figure 6:**
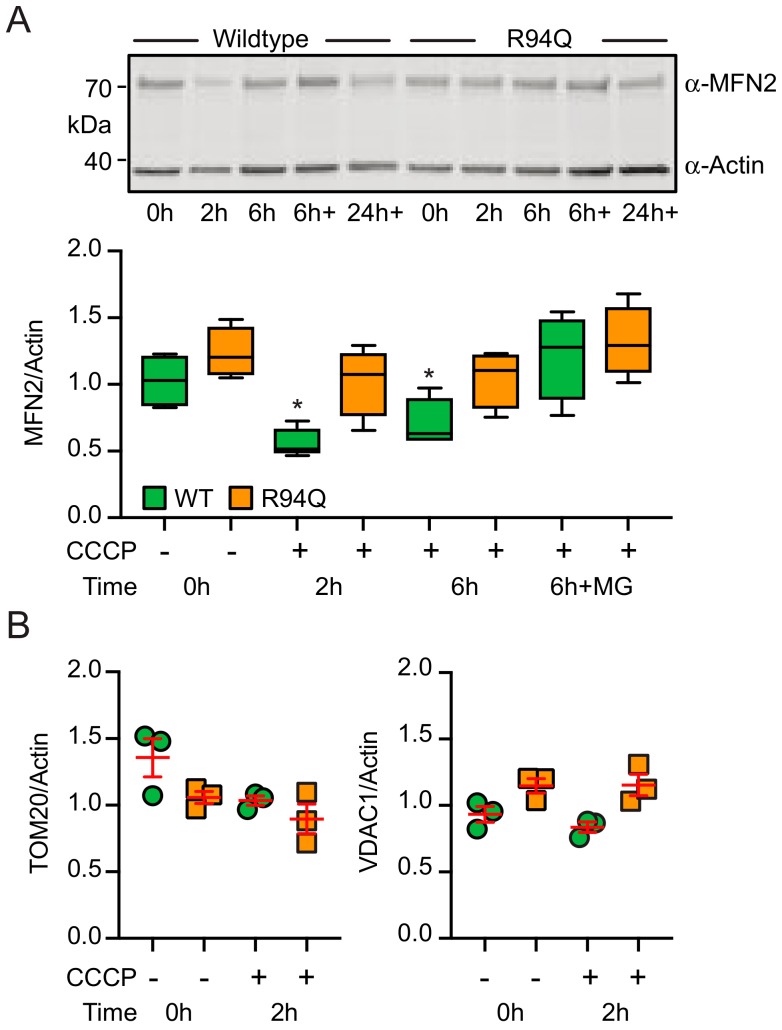
CCCP-induced degradation of MFN2 in R94Q cells. (**A**) Cells were treated with the uncoupler CCCP for the indicated time and immunoblotted against MFN2 in (**A**) and against TOM20 or VDAC1 in (**B**). Actin served as loading control. Size is indicated. Statistical variation is shown in (**A**) as Tukey boxplots and significance calculated using one-way ANOVA and Tukey’s multiple comparisons test, * *p* < 0.05 with n = 5 independent experiments and in (**B**) as individual data points, mean ± SEM.

Therefore, R94Q cells exposed to oxidative stress take up more glucose and ramp up TCA activity but produce less ATP suggesting that these mitochondria are less efficient. We therefore considered a defect in mitophagy, a mitochondrial quality control system mediated by the proteins PINK1 and Parkin, as a possible explanation. Faulty removal of unhealthy mitochondria would probably lead to mitochondria with less efficient coupling between respiration and ATP production. In healthy mitochondria the protein PINK1 is continuously imported and rapidly degraded [27]. When PINK1 import is stalled in unfit mitochondria, enough PINK1 kinase activity is present at the outer mitochondrial membrane to stimulate the translocation of the E3 ubiquitin ligase Parkin to these mitochondria [28]. Mitochondrial Parkin then ubiquitylates outer mitochondrial membrane proteins which ultimately leads to degradation of these mitochondria via autophagosomes, a process called mitophagy. MFN2 was repeatedly identified as being a Parkin target by unbiased methods. In our cells, dissipation of the mitochondrial membrane potential with the uncoupler CCCP—which activates Parkin by increasing endogenous PINK1 protein—indeed resulted in ubiquitination and subsequent degradation of WT but less of R94Q MFN2 (Figure 6a). This effect was most pronounced 2 h after CCCP addition and had a similar trend for the Parkin target TOM2 whereas VDAC1 was not down-regulated at this time point (Figure 6b). These results suggested a defect in the removal of unfit mitochondria.

**Figure 7:**
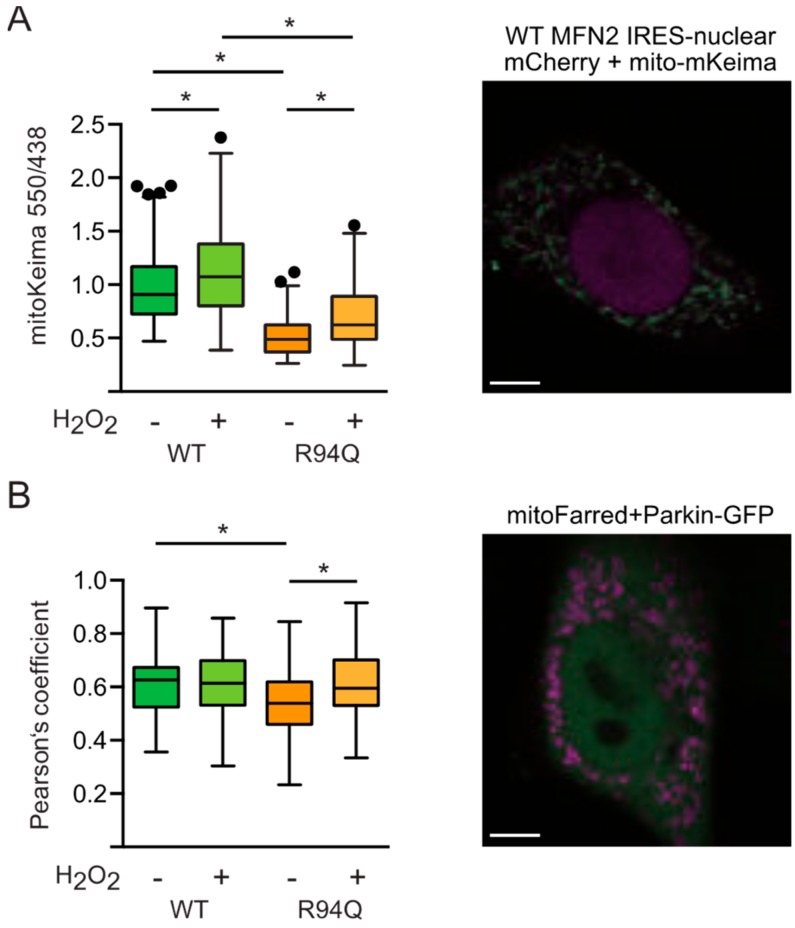
Steady-state mitophagy and mitochondrial Parkin localization in R94Q cells. (**A**) Cells were transfected with mitochondrially targeted mKeima and fluorescence at 620 nm quantified after excitation at 550 nm (red) and 438 nm (green) by confocal microscopy. Scale bar is 5 µm. (**B**) Cells were transfected with Parkin-GFP and mitochondrially targeted TurboFarRed and the fluorescence intensity measured at 488/518 (ex/em) for GFP-Parkin and at 633/640 (ex/em) for Mito-TurboFarRed. Scale bar is 5 µm. Parkin-Mito-TurboFarRed colocalization was quantitated using the JACoP plugin of ImageJ and is expressed as Pearson’s coefficient. Statistical variation is shown as Tukey boxplots and significance calculated using one-way ANOVA and Tukey’s multiple comparisons test, * *p* <0.05, in (**A**) n = 5 independent experiments, in (**B**,**C**) n = 3 independent experiments with a total of (**B**) 91–115 and (**C**) 59–61 individual cells.

This was then reproduced using the genetically encoded reporter mitoKeima [29] which can be used to quantitate the amount of mitochondria being removed by lysosomal degradation. We found less lysosomal mitochondria in R94Q-expressing cells at steady-state conditions which could be increased by treatment with hydrogen peroxide but still not to the levels observed in untreated WT cells (Figure 7a). The same picture emerged when we quantitated the basal colocalization between Parkin-GFP and mitochondria (Figure 7b). We therefore conclude that the R94Q mutation in MFN2 results in a less efficient mitochondrial quality control leading to uncoupled mitochondrial respiration and ATP production in conditions of mild oxidative stress.

## 4. Discussion

We here investigated how a mutation in MFN2 that causes neuronal degeneration of peripheral motoneurons in the hereditary polyneuropathy CMT2A alters key characteristics of mitochondria-like shape, respiration and ATP generation under optimal culture conditions and after a single exposure to mild oxidative stress. Interestingly, under optimal culture conditions only mitochondrial shape was affected by the mutation; R94Q cells had more fragmented mitochondria at baseline. The additional challenge with a low dose of hydrogen peroxide however unmasked additional and probably relevant changes of respiration and mitochondrial ATP generation. Oxidative challenge triggered the mitochondria of cells expressing the R94Q variant of MFN2 but not wildtype to produce less ATP despite an increased oxygen consumption which coincided with an even more fragmented mitochondrial shape. This additional stress thus caused these mutant mitochondria to behave like cells completely lacking MFN2 where mitochondrial respiration was also found to be increased (see Figure 1c and data shown previously [7,8]). It needs to be pointed out, however, that others reported a reduced mitochondrial membrane potential, cellular respiration and leak after down-regulation by antisense oligonucleotides in myoblasts [5] or in heart mitochondria obtained from MFN2−/− mice. This is possibly because these studies were done in skeletal and heart muscle, in tissue and cells, respectively. Interestingly, the cysteine switch C684A which seems to be implicated in mitochondrial hyperfusion, a cellular stress response program, does not mediate the response to oxidative stress in this case as experiments exposing mitochondria to glutathione which attenuates respiration in C684A cells [8] had no effect in the CMT2A-R94Q cells. However, oxidative signaling does not only result in direct modification of so-called thiol switches, reversible modifications of cysteine thiols that play a key role in redox signaling and regulation [30,31], but can also generate second messengers such as 4-hydroxynonenal (HNE), an amphipathic molecule that is generated in response to lipid oxidation which can covalently modify residues in many proteins in different cellular compartments including mitochondria [32]. An effect of HNE on MFN2 has however not been reported yet.

Using mice expressing the R94Q mutation in neurons—of course a much better model of the disease—others observed a combined defect of mitochondrial complexes II and V associated with a drastic decrease of ATP synthesis caused by succinate oxidation [15]. Interestingly, these changes could be reversed by the inhibition of mitochondrial ATP-sensitive potassium channels with 5-hydroxydecanoate [15]. Maybe the hydrogen peroxide challenge affected succinate oxidation. Others also reported that R94Q mitochondria failed to up-regulate ATP production following burst neuronal activation [16]. In wildtype neurons, a first burst generated a H_2_O_2_ but no ATP peak and a second burst applied 30 min later generated a second H_2_O_2_ peak followed by an ATP rise 20 min later. In neurons from R94Q mice, this ATP peak was greatly attenuated [16] which is in line with our findings that prior H_2_O_2_ exposure affects ATP production.

In our cells, the mitochondrial uncoupling of ATP generation and respiration triggered by mild oxidative stress coincided with an altered mitochondrial quality control. We observed a less efficient degradation of MFN2 upon membrane dissipation which is generally attributed to the PINK1/Parkin pathway and less Parkin colocalization with mitochondria under steady-state conditions. It was previously shown that PINK1 phosphorylates MFN2 and promotes Parkin-mediated ubiquitination and subsequent degradation [33]. In these experiments, accumulation of morphologically and functionally abnormal mitochondria in MFN2-deficient MEFs induced respiratory dysfunction [33] similar to our observations. Interestingly, loss of Beclin-1, an important autophagy protein involved in autophagosome formation and maturation, inhibits CCCP-induced Parkin translocation to mitochondria and MFN2 ubiquitination and degradation [34]. Surprisingly, Beclin-1 depletion also rescued the suppression of mitochondrial fusion in MFN2-deficient cells [34] suggesting that MFN2 mutation may somehow affect this pathway although this is difficult to fathom. Alternatively, the mutation could affect phosphorylation of MFN2 by PINK1 required for Parkin binding and mitochondrial Parkin translocation which suppresses mitophagy, however, without impairing mitochondrial fusion [35]. It must be mentioned that others found enhanced, thus not decreased, mitophagy in motoneurons from induced pluripotent stem cells obtained from patients with CMT2A. This observation was accompanied by global reduction in mitochondrial content and changes in mitochondrial positioning without significant differences in survival and axon elongation [36]. These patient-derived neurons show an increased expression of PINK1, PARK2, BNIP3, and a splice variant of BECN1 that appears to be a trigger for mitochondrial autophagic removal [36]. It is possible that this splice variant is only expressed in human motoneurons thus explaining the opposite findings. This view is strengthened by the findings that MFN2 deficiency in mouse muscle reduced autophagy and impaired mitochondrial quality thus contributing to an exacerbated age-related mitochondrial dysfunction [37]. Similar to our findings where the R94Q cells had less reactive oxygen levels under optimal conditions, these authors found that aging-induced Mfn2 deficiency triggers a ROS-dependent adaptive signaling pathway by induction of the HIF1α transcription factor and BNIP3 which may compensate for the loss of mitochondrial autophagy and thereby protect mitochondria [37]. Yet others proposed that different levels of MFN1, a close homologue of MFN2, could alter the effect of mutant R94Q MFN2 on Parkin-mediated mitochondrial degradation indicating that augmentation of MFN1 in the nervous system could be a viable therapeutic strategy for CMT disease [38].
